# Nitric Oxide from IFNγ-Primed Macrophages Modulates the Antimicrobial Activity of β-Lactams against the Intracellular Pathogens *Burkholderia pseudomallei* and Nontyphoidal *Salmonella*

**DOI:** 10.1371/journal.pntd.0003079

**Published:** 2014-08-14

**Authors:** Jessica Jones-Carson, Adrienne E. Zweifel, Timothy Tapscott, Chad Austin, Joseph M. Brown, Kenneth L. Jones, Martin I. Voskuil, Andrés Vázquez-Torres

**Affiliations:** 1 Department of Immunology and Microbiology, University of Colorado School of Medicine, Aurora, Colorado, United States of America; 2 Division of Infectious Diseases, University of Colorado School of Medicine, Aurora, Colorado, United States of America; 3 Department of Biochemistry and Molecular Genetics, University of Colorado School of Medicine, Aurora, Colorado, United States of America; 4 Veterans Affairs Eastern Colorado Health Care System, Denver, Colorado, United States of America; University of Tennessee, United States of America

## Abstract

Our investigations show that nonlethal concentrations of nitric oxide (NO) abrogate the antibiotic activity of β-lactam antibiotics against *Burkholderia pseudomallei, Escherichia coli* and nontyphoidal *Salmonella enterica* serovar Typhimurium. NO protects *B. pseudomallei* already exposed to β-lactams, suggesting that this diatomic radical tolerizes bacteria against the antimicrobial activity of this important class of antibiotics. The concentrations of NO that elicit antibiotic tolerance repress consumption of oxygen (O_2_), while stimulating hydrogen peroxide (H_2_O_2_) synthesis. Transposon insertions in genes encoding cytochrome *c* oxidase-related functions and molybdenum assimilation confer *B. pseudomallei* a selective advantage against the antimicrobial activity of the β-lactam antibiotic imipenem. Cumulatively, these data support a model by which NO induces antibiotic tolerance through the inhibition of the electron transport chain, rather than by potentiating antioxidant defenses as previously proposed. Accordingly, pharmacological inhibition of terminal oxidases and nitrate reductases tolerizes aerobic and anaerobic bacteria to β-lactams. The degree of NO-induced β-lactam antibiotic tolerance seems to be inversely proportional to the proton motive force (PMF), and thus the dissipation of ΔH^+^ and ΔΨ electrochemical gradients of the PMF prevents β-lactam-mediated killing. According to this model, NO generated by IFNγ-primed macrophages protects intracellular *Salmonella* against imipenem. On the other hand, sublethal concentrations of imipenem potentiate the killing of *B. pseudomallei* by NO generated enzymatically from IFNγ-primed macrophages. Our investigations indicate that NO modulates the antimicrobial activity of β-lactam antibiotics.

## Author Summary

β-lactam drugs that inhibit peptidoglycan biosynthesis are often used in the treatment of bacterial infections, including melioidosis. Independent of their antibiotic activity, we have noted that submicromolar concentrations of β-lactams potentiate the killing of intracellular *B. pseudomallei* supported by NO generated by IFNγ-primed macrophages. The production of NO can nonetheless be a double-edged sword, as indicated by our observations that sublethal concentrations of nitric oxide (NO), a diatomic radical produced by phylogenetically diverse organisms to regulate neurotransmission, vascular tone and host defense, tolerize *B. pseudomallei*, nontyphoidal *Salmonella* and *E. coli* against the antimicrobial activity of β-lactams. Accordingly, NO produced in the inflammatory response of macrophages protects nontyphoidal *Salmonella* against β-lactam antibiotics. NO mediates bacterial tolerance to β-lactam antibiotics by inhibiting the electrochemical gradient supported by terminal cytochrome oxidases of the respiratory chain, rather than by decreasing oxidative stress as previously thought.

## Introduction

*B. pseudomallei* are endemic in tropical areas of Southeast Asia, Northern Australia and equatorial countries [1]. This Gram-negative, opportunistic pathogen is a saprophyte that inhabits water and soil, becoming infectious to humans and animals if inoculated through cutaneous abrasions, ingested in contaminated food and water, or inhaled through the respiratory mucosa. Melioidosis can present as an acute, chronic or latent infection [2]. Pneumonia accounts for about 50% of all the cases of *B. pseudomallei* infection [3], [4], whereas septic shock, often a fulminant complication of septicemia, kills 40% of melioidosis patients receiving therapy and 95% of those untreated.

Despite recent advances in antibacterial therapy, management of melioidosis remains a challenge [4]. Antibacterial treatment of melioidosis often spans 20 weeks and requires combined antibiotic therapy. Ceftazidime is often used in the intensive phase, whereas trimethoprim-sulfamethoxazole (TMP-SMX) is used during the eradication phase of treatment [5]. Regardless of intense and vigorous treatment regimes, about 10% of melioidosis patients suffer from relapses [6]. *B. pseudomallei* are intrinsically resistant to most classes of antibacterials [7]. For example, *B. pseudomallei* growing in biofilms are phenotypically tolerant to doxicycline, ceftazidime, imipenem and TMP-SMX [8], [9]. The efflux pumps BpeAB-OprB, BpeEF-OprC and AmrAB-OprA further increase the resistance of this opportunistic pathogen to β-lactams, aminoglycosides, macrolides, fluoroquinolones, chloramphenicol and polymyxins [10]–[12]. Class A and D β-lactamases add to the arsenal of enzymatic systems that protect *B. pseudomallei* against ampicillin, carbenicillin, ceftazidime and imipenem [13]–[15]. In addition to these well-characterized mechanisms of antibiotic resistance, changes in bacterial physiology in response to host environmental conditions may promote resistance to antibiotics. For example, anaerobiosis, which is normally attained in the hepatic, splenic and prostate abscesses of melioidosis patients, induces a population of *B. pseudomallei* remarkably refractory to several classes of clinically important antibacterials [16].

In addition to being an intrinsic component of the antimicrobial arsenal of vertebrate hosts [17], the signaling properties of NO have been co-opted by prokaryotic and eukaryotic organisms. NO produced endogenously by bacterial NO synthase protects *Bacillus subtilis* against a wide spectrum of antibiotics [18]. This adaptive response of *Bacillus* might lessen the bactericidal activity of antibiotics produced by saprophytic microorganisms populating the soil. Modification of drugs and potentiation of antioxidant defenses have been evoked as mechanisms underlying the NO-induced antibiotic resistance of *Bacillus* [18]. NO produced in the inflammatory response has also been shown to shield Gram-positive and –negative bacteria against the antimicrobial activity of bactericidal antibiotics. *Salmonella enterica* survives exposure to members of the aminoglycoside family in response to the NO generated intracellularly by IFNγ-activated macrophages [19], a situation that had previously been noted for *Listeria* with ampicillin [20]. Given the recently described role of NO in inducing resistance of phylogenetically diverse bacteria to different classes of antibiotics and the recent controversy attributing oxidative stress as the mechanism of action of bactericidal antibiotics [18], [21]–, we tested whether NO generated chemically or enzymatically modifies the antimicrobial activity of β-lactams against *B. pseudomallei* and two representative members of the enterobacteriaceae family.

## Methods

### Bacterial strains and growth conditions

Strain K96243, a clinical isolate of *B. pseudomallei* [24], was grown in the BSL3 laboratory of the Department of Microbiology at the University of Colorado School of Medicine. This facility has been certified by the CDC for work with select agents. *E. coli* strain 3110 and *S. enterica* serovar Typhimurium strain 14028 s were also used in the course of these investigations. Where indicated, *Salmonella* strains AV0468, AV07140 and AV07141 deficient in the flavohemoprotein *hmp*, acetate kinase *ackA* or phosphotransacetylase *pta*, respectively, were used. The bacteria were grown overnight to stationary phase in LB broth supplemented with 4% (v/v) glycerol (LBG) at 37°C and 315 RPM in a shaker incubator (New Brunswick Innova, Edison, NJ). Where indicated, the bacteria were grown to log phase to OD_600_ of 0.6.

### Susceptibility of *B. pseudomallei* to antibiotics

Log phase *B. pseudomallei* was grown from overnight cultures in a shaker incubator at 37°C in LBG broth to an OD_600_ of 0.6. Log and stationary phase *B. pseudomallei* cultures were diluted to OD_600_ of 0.012 in 1 ml of LBG broth in 14 ml polypropylene tubes containing sterile stirrer magnets. The killing activity of imipenem was assessed in bacterial cultures at the indicated concentrations. The tubes were loosely capped and placed on a magnetic stirrer in a 37°C cell culture incubator. The anti-*B. pseudomallei* activity of ceftazidime was tested in 250 ml flasks as previously described [16]. Both antibiotics were purchased from Sigma-Aldrich, St. Louis, MO. Selected cultures were co-treated with spermine NONOate or DETA NONOate, which generate NO with half-lives of 39 min and 20 h, respectively, at 37°C, pH 7.4. In selected experiments the susceptibility of log phase *E. coli* and *Salmonella* was also tested. Where indicated, *E. coli* and *Salmonella* were grown in EG medium [i.e., E salts (0.2 g/L MgSO_4_, 2 g/L C_6_H_8_O_7_-H_2_O, 10 g/L K_2_HPO_4_, 3.5 g/L Na(NH_4_)HPO_4_-4H_2_O) supplemented with 0.4% glucose]. The number of surviving bacteria after antibiotic treatment was determined after culture on LB agar plates, and the fraction of bacteria that survived antibiotic treatment was calculated as (cfu t_n_/cfu t_0_)×100.

### Construction of the transposon mutant pool and deep-sequencing analysis

A mini-Mariner transposon [25], [26] expressed from the suicide plasmid pTBurk1 was electroporated into *B. pseudomallei* strain K96243. This plasmid contains the *Himar1* transposable element with a kanamycin cassette flanked by inverted repeats (IR). The *Himar1* transposase was chosen because of its TA dinucleotide specificity. Bacteria with an integrated kanamycin cassette were selected on LB agar plates containing 50 µg/ml kanamycin and 100 µg/ml zeocin. The sequencing libraries were quantified using the Agilent Bioanalyzer DNA7500 chip, multiplexed, cluster amplified, and sequenced on the Illumina MiSeq platform. Sequencing reads containing the *Himar1* IR sequence and the adjacent TA were isolated from the raw fastq file. The IR sequence was removed from the analysis. The processed reads were mapped onto the *B. pseudomallei* K96243 reference genome using the program Bowtie2 with local alignment settings and a *k* value of 1 [27]. Annotation of TA sites was accomplished using seqanno, a custom set of Python scripts (https://github.com/brwnj/seqanno) used to characterize specific genomic sequences. The publicly available code was used to quantify reads over a given sequence, annotate those counts at the gene level, compare results between samples, and annotate using a UniProt flat file for *B. pseudomallei*.

### Determination of endogenous H_2_O_2_ synthesis

*B. pseudomallei* grown overnight in LBG broth were subcultured 1∶100 in LBG broth at 37°C with shaking. The generation of H_2_O_2_ by *B. pseudomallei* was measured in stationary phase bacteria diluted to an OD_600_ of 0.5. Selected bacterial cultures were treated for 1 h at 37°C with 12.5 µg/ml imipenem in the presence or absence of 100 µM spermine NONOate or 500 µM KCN. The cultures were continuously agitated with a magnetic stir-bar. The specimens were placed into a sealed, temperature-controlled chamber (World Precision Instruments, Inc., Sarasota, FL) containing a small magnetic stir bar. To prevent artifacts associated with a possible loss of viability, the experiments were carried out for 1 h after exposure to imipenem before the onset of killing took place. The H_2_O_2_ accumulated in the bacterial cultures after 1 h incubation was measured pollarographically for about 2 min using an ISO-H_2_O_2_ sensor attached to an APOLLO 4000 free radical analyzer (World Precision Instruments). The concentration of H_2_O_2_ produced by the bacterial cultures was calculated by regression analysis of a standard curve generated with known concentrations of H_2_O_2_.

### O_2_ consumption

*B. pseudomallei* was grown overnight in LBG broth at 37°C with shaking. Overnight cultures were diluted to OD_600_ of 0.5 in a volume of 1 ml. Where indicated, 100 µM spermine NONOate or 500 µM KCN were added to the bacterial cultures. The samples were transferred to a multiport temperature-controlled chamber, and the consumption of O_2_ by the bacteria was measured over a 5 min period using an ISO-OXY-2 O_2_ sensor attached to an APOLLO 4000 free radical analyzer. To ensure the even distribution of gases in the chamber, the samples were placed on a chamber containing a magnetic stir-bar. The data are expressed as µM of O_2_.

### Nitrate reductase activity

Nitrate reductase enzymatic activity was monitored by measuring the accumulation of NO_2_^−^ in the cultures. Bacterial pellets of *B. pseudomallei* grown overnight in LBG broth diluted to an OD_600_ of 0.6 were moved into the anaerobic chamber, where they are aliquoted into 1 ml volumes in LBG broth or LBG broth supplemented with 50 mM NaNO_3_^−^. The O_2_ in the LBG broth had been eliminated by culturing the media in the anaerobic chamber for at least 24 h. The bacterial cells were allowed to reduce NO_3_^−^ to NO_2_^−^ for 2.5 h. NO_2_^−^ concentrations were measured spectrophotometrically at 550 nm after mixing with an equal volume of Griess reagent (0.5% sulfanilamide and 0.05% *N*-1-naphthylethylenediamide hydrochloride in 2.5% phosphoric acid). NO_2_^−^ concentrations were calculated by regression analysis using standard curves prepared with NaNO_2_.

### Proton motive force

The membrane potential of *S*. Typhimurium grown in LB broth and EG medium to OD_600_ of 0.5 was measured with the fluorescent probe DiSC_3_(5) (Molecular Probes, Eugene, OR). The pellet of 1 mL of cells grown to log phase in LB broth or EG medium was resuspended in 5 mM HEPES, pH 7.2, supplemented with 5 mM casamino acids or 5 mM glucose, respectively. Samples were treated in 1 ml aliquots with 750 µM spermine NONOate for 15 minutes at 37°C. DiSC_3_(5) was added to a final concentration of 1 µM from a stock solution made in DMSO. DiSC_3_(5) was allowed to equilibrate in the cells before fluorescence measurements were collected in a Synergy 2 microtiter plate reader (BioTek, Winooski, VT) using excitation and emission wavelengths of 590 and 680 nm, respectively.

### Macrophage assays

J774 murine macrophage-like cells (clone ATCC TIB-67) were grown in RPMI medium supplemented with 10% fetal bovine serum (BioWhittaker, Walkersville, MD), 15 mM Hepes, 2 mM L-glutamine, 1 mM sodium pyruvate (Sigma-Aldrich, St. Louis, MO), and 100 U·ml^−1^/100 mg·ml^−1^ of penicillin/streptomycin (Cellgro). The macrophages were treated with 200 U/ml of recombinant murine IFNγ (Peprotech, Rocky Hill, NJ) 16 h before infection. The macrophages were infected for 2.5 h with *B. pseudomallei* at an MOI of 4, after which the media was exchanged with RPMI^+^ containing 350 µg/ml kanamycin. One hour later, *B. pseudomallei*-infected cells were incubated for 4 h in fresh culture media containing 250 µg/ml kanamycin. The media was then replaced with fresh media containing increasing concentrations of imipenem in the presence or absence of 500 µM of the iNOS inhibitor aminoguanidine. In parallel experiments, macrophages were infected for 25 min with *S.* Typhimurium at an MOI of 2. Extracellular *Salmonella* were killed after treatment for 1 h with 50 µg/ml gentamicin. The *Salmonella*-infected macrophages were then incubated in fresh RPMI media containing 10 µg/ml gentamicin in the presence or absence of aminoguanidine, which was maintained in the culture media for the rest of the experiment. After 8 h, the *Salmonella*-infected cells were washed and fresh media containing imipenem was added to the cultures. The *B. pseudomallei* and *Salmonella* burden in the cultures was determined 12–14 h after exposure to imipenem. The amount of nitrite, a terminal oxidative product of NO, synthesized by the macrophages was estimated by the Griess reaction.

### Statistical analysis

The data were analyzed using a Student's paired *t* test. Determination of statistical significance between multiple comparisons was achieved using one-way analysis of variance (ANOVA) followed by a Bonferroni post-test. Data were considered statistically significant when *p*<0.05.

## Results

### NO protects *B. pseudomallei* from antibiotics that target peptidoglycan biosynthesis

**Figure 1:**
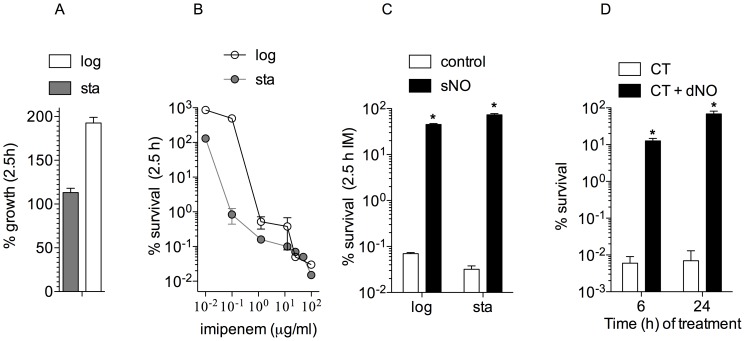
Effect of NO on the anti-*B. pseudomallei* activity of β-lactam antibiotics. Growth of log and stationary phase *B. pseudomallei* in LBG broth 2.5 h after culture (A). *B. pseudomallei* grown to log (log) or stationary (sta) phase were treated for 2.5 h with increasing concentrations of imipenem (B). Panel C shows the effect of 100 µM spermine NONOate (sNO) on the anti-*B. pseudomallei* activity of 25 µg/ml imipenem (IM). The protection afforded by 2.5 mM DETA NONOate (dNO) against 64 µg/ml ceftazidime (CT) is shown in D. The data are the mean ± SD from 3 observations collected in 2 separate days. *p*<0.001 compared to the antibiotic-treated group.

The β-lactam antibiotic imipenem has been used in the clinic to treat people with melioidosis [7], [28]. Under the experimental conditions tested, stationary *B. pseudomallei* did not grow 2.5 h after subculture in LB broth supplemented with 4% glycerol (figure 1A). Despite this lack of growth, 1 µg/ml imipenem reduced the viability of stationary phase *B. pseudomallei* by ∼1,000-fold (figure 1B). These findings contrast with those reported earlier by Eng *et al* who found poor antimicrobial activity of imipenem against nongrowing bacteria [29]. Differences in bacterial species might account for these discrepancies. As expected, imipenem effectively killed log phase *B. pseudomallei* (figure 1B), a population that double in numbers 2.5 h after culture in fresh LBG broth (figure 1A). Together, our investigations indicate that imipenem can be equally efficient at killing both replicating and non-replicating *B. pseudomallei*. Our investigations also indicate that the imipenem-dependent inhibition of both peptidoglycan remodeling in stationary phase bacteria and *de novo* peptidoglycan biosynthesis in growing *B. pseudomallei* can exert profound antimicrobial activity. The NO donor spermine NONOate, which has a half-life of 39 min, was used to test whether this diatomic radical abrogates the antimicrobial activity of imipenem. Given that *B. pseudomallei* is extraordinarily susceptible to the antimicrobial activity of NO [30], spermine NONOate was titrated in order to find conditions in which the viability of *B. pseudomallei* was not affected upon NO treatment. The addition of 100–200 µM spermine NONOate, which generates 2 moles of NO per mole of parent compound, failed to kill *B. pseudomallei* under the experimental conditions used in the course of these investigations. The concentrations of NO used in these experiments effectively inhibited growth of log phase *B. pseudomallei* (not shown). The addition of 100 µM spermine NONOate completely abrogated killing by imipenem against both stationary and log phase *B. pseudomallei* (figure 1C). We noticed that the colonies of *B. pseudomallei* treated simultaneously with imipenem and NO took even longer to grow that those of NO-treated controls, indicating that NO does not prevent imipenem from poisoning penicillin-binding proteins in the cell wall. The protective effects appear to be explained by the NO released by spermine NONOate and not the polyamine base, since spermine did not affect the imipenem-dependent killing of *B. pseudomallei* (not shown).

We also tested the effects of NO on the anti-*B. pseudomallei* activity of ceftazidime, which is the β-lactam antibiotic of choice in the acute phase of treatment of melioidosis [31]. Ceftazidime failed to kill *B. pseudomallei* under the same experimental conditions under which imipenem exerted profound bactericidal activity (not shown). Therefore we adopted a system of long-term exposure to a high concentration of ceftazidime that has been shown to sustain cytotoxicity against the seemingly resistant strain of *B. pseudomallei* used in our studies [16]. *B. pseudomallei* was effectively killed 6 h after the addition of 64 µg/ml ceftazidime (figure 1D). We used this *in vitro* culture system to test the effects of NO on ceftazidime-mediated killing of *B. pseudomallei*. To ensure long-term release of NO, these investigations made use of the slow NO donor DETA NONOate, which has an estimated half-life of 20 h at 37°C, pH 7.4. The addition of 2.5 mM DETA NONOate abrogated most of the anti-*B. pseudomallei* activity associated with ceftazidime treatment (figure 1D). Together, these findings indicate that chemically-generated NO protects *B. pseudomallei* against β-lactam antibiotics.

### NO induces antibiotic tolerance

**Figure 2:**
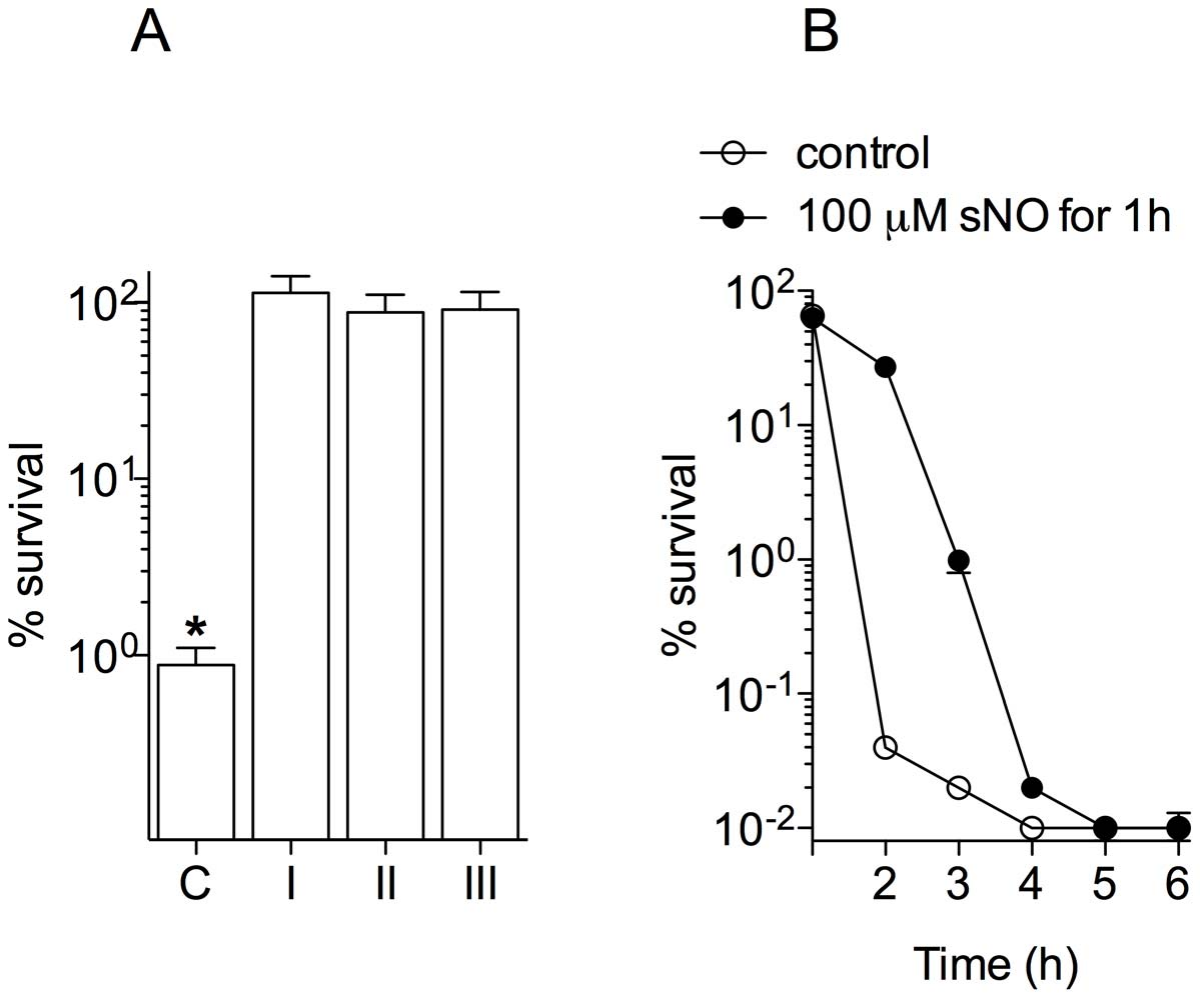
NO tolerizes *B. pseudomallei* to the antimicrobial activity of imipenem. (A) All bacteria in groups I-III were treated with 25 µg/ml imipenem for 2.5 h. *B. pseudomallei* in group I were treated with 100 µM spermine NONOate (sNO) 1 h after exposure to imipenem; sNO was removed from group II after 1 h of treatment; sNO was maintained in group III during the 2.5 h of challenge. The survival of control bacteria (C) treated with 25 µg/ml of imipenem is shown for comparison. Panel B shows the fraction of *B. pseudomallei* that survived 25 µg/ml imipenem at the indicated times. Selected specimens (dark symbols) were treated with 100 µM sNO during the first hour of exposure to imipenem. The data are the mean ± SEM from 5 observations collected on 2 separate days. *p*<0.001 compared to groups I–III.

The following experiments were performed in order to determine whether NO tolerizes *Burkholderia* against the cytotoxic actions of antibiotics or stimulates long-lasting genetic resistance. Imipenem was used to test these two models because 1) this β-lactam antibiotic is endowed with potent anti-*Burkholderia* activity, 2) imipenem-mediated killing occurs within a few hours of exposure, and 3) NO induces excellent protection against this drug. Two independent experimental approaches tested whether NO tolerizes *Burkholderia* or induces long-lasting genetic resistance. First, 100 µM spermine NONOate was added to the cultures 1 h after exposure to 25 µg/ml imipenem. As seen with *Burkholderia* co-exposed to NO and imipenem, the addition of NO 1 h after imipenem treatment abrogated killing of stationary phase *B. pseudomallei* (figure 2A). Second, bacterial cultures were pretreated with spermine NONOate and imipenem for 1 h and then washed by centrifugation. The bacterial cells were then resuspended with fresh LBG broth containing 25 µg/ml imipenem. Again, these cultures were as protected as cultures receiving NO and imipenem during the full 2.5 h of challenge. These findings indicate that the protective actions afforded by NO are immediate and can occur after the bacteria have been exposed to imipenem. To test the duration of the protective effects associated with NO treatment, *B. pseudomallei* were treated with NO for 1 h, and then placed in fresh media containing 12.5 µg/ml imipenem for up to 5 additional hours. The protective effects associated with NO treatment were lost over time (figure 2B). For instance, imipenem killed more than 99.99% of the bacteria in the population 5 h after NO was removed from the cultures. Cumulatively, our investigations indicate that the protective effects afforded by NO against imipenem are transitory and are best observed in cells actively undergoing nitrosative stress.

### Identification of loci associated with NO-induced tolerance to imipenem

To identify loci that may be associated with the NO-induced tolerance to imipenem, a *B. pseudomallei* mutant library was constructed using a mini-mariner transposon with an insertion specificity for a TA dinucleotide [25], [26]. Sequencing of genomic DNA at the transposon-chromosome junctions allowed us to determine the coverage of the library. Overall, the library consists of 35,075 independent clones encompassing 28,543 and 6,532 intragenic and intergenic insertions, respectively. On average, each gene harbors 3–4 independent transposons. Disrupted genes were defined as those that sustained 6 or more sequence-reads per site within the internal 5–80% of gene length. Essential genes in *Burkholderia* were defined as those sustaining fewer than 6 sequence-reads within 3–97% of the open reading frame length. We estimate that 590 genes are essential for growth of *B. pseudomallei* under the experimental conditions tested (table S1). The estimated essential genes encode functions such as DNA replication, chromosome maintenance, lipid metabolism, translation, cell division, and energy and nucleic acid metabolism (figure S1).

We used this transposon library to identify transposon mutants with increased resistance to imipenem. The transposon library was treated for 2.5 h with 12.5 µg/ml imipenem and/or 750 µM spermine NONOate at an OD_600_ of 0.5 in LBG broth. The specimens were then subcultured in 25 ml of fresh LBG broth until the bacteria reached an OD_600_ of 0.6. The frequency of transposons in genomic DNA isolated from *B. pseudomallei* treated with either spermine NONOate, or spermine NONOate and imipenem was quantified by Illumina deep-sequencing as described previously for *Heamophilus* [32]. Sequencing data from 3 independent experiments were averaged, the fold change between samples calculated, and false discovery rate analysis determined. Sixteen genes with several transposons were found to be enriched in the group treated with imipenem and spermine NONOate (table 1). Among the positively selected genes were mutants with transposons in loci encoding cytochrome c oxidase function. In addition, the *moaC* and *mogA* genes involved in molybdenum utilization were also positively selected. Molybdenum is a common cofactor of enzymes such as nitrate reductases that allow bacteria to grow using NO_3_^−^ for respiration. LBG broth and the autoxidation of the NO generated from spermine NONOate are likely sources of the terminal electron acceptor NO_3_^−^ in our system. Together, the positive selection of clones bearing mutations in cytochromes and molybdenum utilization genes suggest that disruption of the electron transport chain provides *B. pseudomallei* a selective advantage against the killing of imipenem. In addition, the disruption of several genes associated with nucleotide metabolism, tRNA synthesis, β-lactamase processing and transcriptional regulation appear to provide a selective advantage to *B. pseudomallei* against the antimicrobial activity of imipenem.

**Table 1:** Positively selected genes bearing transposon mutations that increase the tolerance of *B. pseudomallei* to imipemen.

Genes	Description	Fold change^1^
BPSL0453	cytochrome *c* oxidase	3.25
BPSL3181	cytochrome *c*	4.37
*moaC*	molybdenum cofactor biosynthesis protein	6.06
*mogA*	molybdenum cofactor biosynthesis protein	3.03
*oxa*	β-lactamase precursor	8.09
*obgE*	GTPase	6.67
*amn*	AMP nucleosidase	3.10
*gmk*	guanylate kinase	7.68
*glnB1*	nitrogen regulatory protein P-II 1	4.59
*trmB*	tRNA (guanine-N-(7)-)methyltransferase	4.51
*clpA*	ATP-dependent Clp protease	3.53
BPSS1056	CopG Family transcriptional regulator	8.12
BPSL3313	HNS-like transcriptional regulator	3.70
BPSL0428	Hypothetical protein	34.00
BPSL1511	Hypothetical protein	4.08
BPSL2413	Hypothetical protein	13.12

1 The fold change represents the ratio of the number of sequence reads in the imipenem + spermine NONOate sample over spermine NONOate control.

### Inhibition of bacterial respiration protects *B. pseudomallei* against imipenem

**Figure 3:**
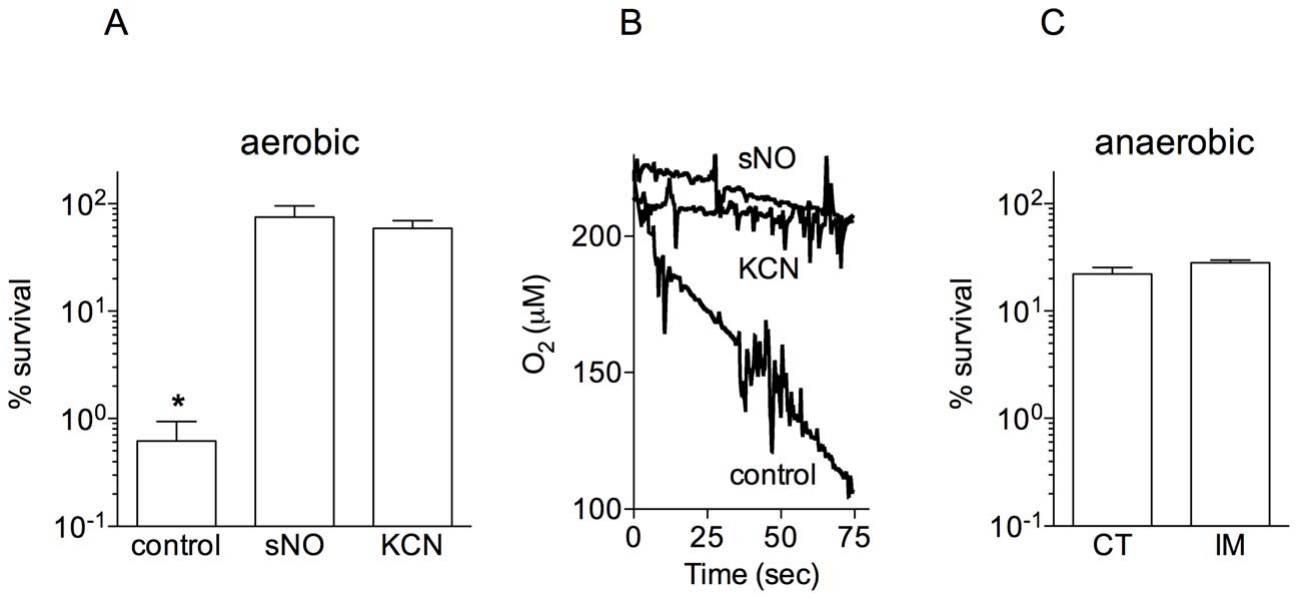
Respiratory activity of *B. pseudomallei* undergoing nitrosative stress. Survival of *B. pseudomallei* after 2.5 h of treatment with 12.5 µg/ml of imipenem (A). Some of the specimens were co-treated with either 500 µM potassium cyanide (KCN) or 100 µM spermine NONOate (sNO). The percent survival was determined as described in figure 1. The data are the mean ± SD from 3 observations collected on 3 separate days. The ability of *B. pseudomallei* to consume O_2_ was monitored polarographically (B). The data shown in panel B are representative from 3 independent experiments. *B. pseudomallei* were adapted for 5 h in an anaerobic chamber and the % survival scored 2.5 h after the addition of 64 µg/ml ceftazidime (CT) or 25 µg/ml imipenem (IM) (C). The results in C are expressed as % survival over controls that were not exposed to antibiotics. *B. pseudomallei* controls in the anaerobic chamber neither grew nor lost viability in the time frame tested. *p*<0.01 compared to sNO- and KCN-treated groups.

Given the selectivity of NO for metal prosthetic groups in the terminal oxidases of the electron transport chain and the fact that mutations in components of the respiratory chain provided a competitive advantage to *Burkholderia* in response to imipenem (table 1), it is possible that the antibiotic tolerance elicited in response to NO is associated with a loss in respiratory function. To test this hypothesis, we measured whether the addition of classical antagonists of terminal oxidases of the electron transport chain affects the susceptibility of *B. pseudomallei* to imipenem. As seen with NO, the respiratory inhibitor potassium cyanide (KCN) prevented the imipenem-dependent killing of *B. pseudomallei* (figure 3A). To determine whether the concentrations of NO and KCN that protect *B. pseudomallei* against imipenem affect the respiratory activity of *B. pseudomallei*, we measured the consumption of O_2_. Compared to untreated controls grown in LBG broth saturated with O_2_ (figure 3B), bacterial cultures treated with 100 µM spermine NONOate or 500 µM KCN had reduced respiratory activity. Under the conditions tested, neither 100 µM spermine NONOate nor 500 µM KCN killed *B. pseudomallei*. These findings suggest that terminal cytochromes of the electron transport chain are critical molecular targets of NO-induced antibiotic resistance. Our investigations also suggest that β-lactam antibiotics require an active electron transport chain to exert their antimicrobial activity. This model is supported further by the fact that the antimicrobial activity of ceftizidime and imipenem was dramatically reduced in anaerobic cultures (figure 3C).

### Inhibitors of PMF prevent imipenem-mediated killing

**Figure 4:**
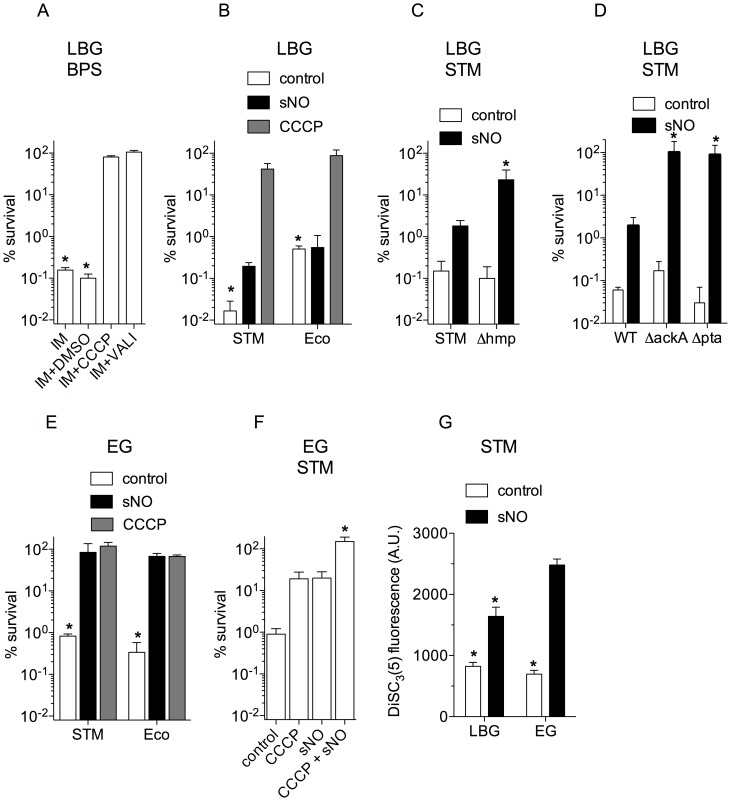
Effect of the PMF on the antimicrobial activity of imipenem. Effect of 50 µM CCCP or 25 µM valinomycin on the anti-*Burkholderia* activity of 12.5 µg/ml imipenem (IM) (A). Stationary phase *B. pseudomallei* controls grown overnight in LBG broth were treated with 0.25% DMSO for 2.5 h. Killing of *S.* Typhimurium (STM) and *E. coli* (Eco) grown to log phase in LBG broth (B, C, D) or EG medium (E, F) by IM. Selected samples were treated with 750 µM spermine NONOate (sNO) or 50 µM CCCP. The samples in F were treated with 10 µM CCCP ± 250 µM sNO. *p*<0.01 compared to the CCCP, valinomycin- or sNO-treated groups. The PMF was estimated by measuring the accumulation of DiSC_3_(5) in STM (G). The data are expressed as arbitrary fluorescent units (A.U.). *p*<0.001 compared to the sNO-treated EG group. The data are the mean ± SEM from 3–5 independent observations collected on 2 separate days.

The enzymatic activity of terminal cytochrome oxidases and nitrate reductases of the electron transport chain help maintain an electrochemical gradient across the cytoplasmic membrane [33]. It is therefore possible that decreases of PMF in response to NO could mediate antibiotic tolerance. To test this idea, we evaluated the effect that collapsing the PMF has on the anti-*B. pseudomallei* activity of imipenem. The protonophore carbonyl cyanide 3-chlorophenylhydrazone (CCCP) and the ionophore valinomycin were chosen for these investigations, because these drugs dissipate the ΔH^+^ and ΔΨ components of the PMF by facilitating the transport of H^+^ and K^+^ down an electrochemical potential gradient. Remarkably, CCCP and valinomycin protected *B. pseudomallei* against the antimicrobial activity of imipenem (figure 4A), supporting the model that the antimicrobial activity of this β-lactam antibiotic depends on a functional PMF.

Next, we tested whether NO abrogates killing of other Gram-negative bacteria by imipenem. Surprisingly, exposure of *S. enterica* serovar Typhimurium or *E. coli* grown in LBG broth to 750 µM spermine NONOate had a small effect on the antimicrobial activity of imipenem (figure 4B). To investigate whether the failure of NO to protect *Salmonella* against imipenem is associated with antinitrosative defenses, we compared the susceptibility of wild-type and an *hmp* mutant that lacks the main mechanism of NO detoxification known in *Salmonella* and *E. coli* [34], [35]. NO induced remarkable levels of protection against imipenem in *hmp*-deficient *Salmonella* (figure 4C). It should be noted that about 90% of the *hmp* mutants were still killed by imipenem, raising the possibility that, in addition to antinitrosative defenses, the metabolic pliability of enteric bacteria could prevent the protective effects associated with NO. We reasoned that the incomplete protection afforded by NO to *Salmonella* and *E. coli* might be related to the fact that these facultative anaerobes can energize the PMF using alternative electron acceptors. If this were the case, then we would predict that 1) mutations that favor metabolism through the TCA cycle could allow for a more complete NO-induced tolerance to imipenem, 2) classical PMF inhibitors may induce imipenem tolerance under conditions that NO fails to do so, 3) growth of *Salmonella* and *E. coli* with glucose as the sole carbon source might estimulate NO-induced antibiotic tolerance, and 4) NO may have different effects on the PMF according to the carbon source used for growth. The following experiments were performed to test these predictions. 1) We tested *ackA* and *pta* mutants unable to ferment pyruvate to acetate, thus forcing *Salmonella* to more fully utilize the TCA cycle and oxidative phosphorylation. Remarkably, NO completely protected *ackA* and *pta* mutants against the antimicrobial activity of imipenem (figure 4D). 2) In contrast to NO, the addition of 50 µM CCCP protected *Salmonella* and *E. coli* grown in LBG broth against 12.5 µg/ml imipenem (figure 4B), demonstrating that inhibition of PMF induces tolerance to imipenem under conditions that NO is unable to do so. 3) *E. coli* and *Salmonella* grown in E salts medium supplemented with 0.4% glucose (i.e., EG medium) were efficiently killed by imipenem (figure 4E). Moreover, the addition of 750 µM spermine NONOate or 500 µM KCN similarly protected most *Salmonella* and *E. coli* grown in EG medium against the bactericidal activity of 12.5 µg/ml imipenem. *Salmonella* exposed to suboptimal concentrations of spermine NONOate and CCCP (i.e., 250 and 10 µM, respectively) became fully tolerant to imipenem (figure 4F). Lastly, 4) we measured the PMF in the BSL2 pathogen *S.* Typhimurium with 3,3′-dipropylthiadicarbocyanine iodide [DiSC_3_(5)], the fluorescence of which is inversely proportional to the PMF [36]. As expected, valinomycin increased DiSC_3_(5)-mediated fluorescence (figure S2). DiSC_3_(5)-mediated fluorescence increased (*p*<0.001) in both *Salmonella* grown to OD_600_ of 0.5 in LBG broth or EG medium after exposure to 750 µM spermine NONOate (figure 4G), suggesting that NO inhibits the PMF under both conditions. It should be noted, however, that DiSC_3_(5) fluorescence was significantly (*p*<0.001) lower in NO-treated *Salmonella* grown in LBG broth than NO-treated controls grown in EG medium, suggesting that NO is less efficient at inhibiting the PMF in cells grown in LBG broth. Our investigations indicate that the protection afforded by NO against imipenem is dependent on the degree of PMF inhibition. Together, these findings suggest that both metabolic activity and antinitrosative defenses modulate NO-mediated tolerance to imipenem.

### The protective effects of NO cannot be explained by reduced oxidative stress

**Figure 5:**
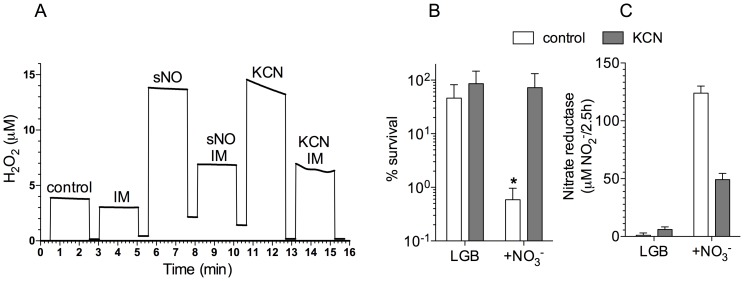
Effect of imipenem and nitrosative stress on H_2_O_2_ synthesis. The production of H_2_O_2_ by *B. pseudomallei* diluted to OD_600_ of 0.5 was measured polarographically (A). Some of the specimens were treated with 12.5 µg/ml imipenem (IM), and where indicated the bacterial cells were treated with 100 µM spermine NONOate (sNO) or 500 µM KCN. Untreated cells are shown as controls. The H_2_O_2_ probe was washed with fresh LB broth between individual specimens. The data are representative of 3 observations collected on 3 separate days. The killing of anaerobic *B. pseudomallei* by 50 µg/ml IM was tested in LBG broth in the presence or absence of 50 mM NO_3_^−^ (B). *p*<0.01 compared to the KCN-treated group. Nitrate reductase activity was monitored by measuring the accumulation of NO_2_^−^ by the Griess reaction (C).

Our investigations demonstrate that NO induces tolerance to β-lactams by collapsing the PMF. It remains possible that the protection afforded by NO against β-lactam antibiotics could emanate from its ability to promote antioxidant defenses [18]. Following this line of reasoning, the limited antimicrobial activity of imipenem against anaerobic *B. pseudomallei* could be interpreted as a sign that oxidative stress is required for killing. Consequently, we tested whether imipenem induces oxidative stress in *B. pseudomallei*, and whether NO affects this response. Membrane soluble H_2_O_2_, which arises by the spontaneous or enzymatic dismutation of O_2_^−^, was used as readout of overall production of reactive oxygen species. *B. pseudomallei* treated with sublethal concentrations of imipenem produced consistently lower concentrations of H_2_O_2_ than untreated controls (figure 5A). The addition of 100 µM spermine NONOate to the bacterial cultures increased the amount of H_2_O_2_ generated by 3-fold. Similar to NO-treated cells, KCN-treated *B. pseudomallei* generated about 15 µM H_2_O_2_. The addition of a sublethal concentration of imipenem significantly (*p*<0.05) reduced the amount of H_2_O_2_ generated by *B. pseudomallei* in response to NO or KCN. These findings are consistent with those reported by Liu and Imlay, who conjectured that the effects of β-lactams on respiration could reflect damage of the cell envelope and dissipation of the back pressure of the proton motive force [22]. Our investigations indicate that NO-mediated antibiotic tolerance cannot be explained by diminished oxidative stress; nor does the imipenem-mediated killing of *B. pseudomallei* appear to be dependent on the elicitation of oxidative stress. Collectively, these data support recent investigations that have questioned oxidative stress as the mode of action of bactericidal antibiotics [22], [23]. Our data also suggest that NO-induced antibiotic resistance takes place independently of its effects on antioxidant defenses.

According to our proposed model that the NO-dependent collapse of the PMF induces tolerance to β-lactams, the poor antibiotic activity of imipenem against anaerobic *B. pseudomallei* could reflect a lack of respiratory activity. To shed light into this possibility, the antimicrobial activity of imipenem was tested in anaerobic *B. pseudomallei* grown in LBG broth supplemented with 50 mM of the terminal electron acceptor NO_3_^−^. Anaerobic cells respiring NO_3_^−^ became susceptible to 50 µg/ml imipenem (figure 5B). We tried to determine the effects of NO treatment on the killing of anaerobic *Burkholderia* by imipenem but, as previously noted [16], anaerobic bacteria were found to be extraordinarily susceptible to 100 µM spermine NONOate. Therefore, KCN was used instead. The addition of KCN abrogated the antibiotic activity of imipenem against anaerobic *B. pseudomallei* cultured in LBG broth supplemented with 50 mM NO_3_^−^ (figure 5B). The concentrations of KCN that elicited protection also inhibited nitrate reductase activity (figure 5C). These findings indicate that imipenem has antibiotic activity in the absence of O_2_ and derived reactive oxygen species if the electron transport chain is energized by the reduction of alternative electron acceptors such as NO_3_^−^.

### Effects of nitric oxide produced by IFNγ-treated macrophages on the antimicrobial activity of imipenem against intracellular *B. pseudomallei* and *S. enterica*

**Figure 6:**
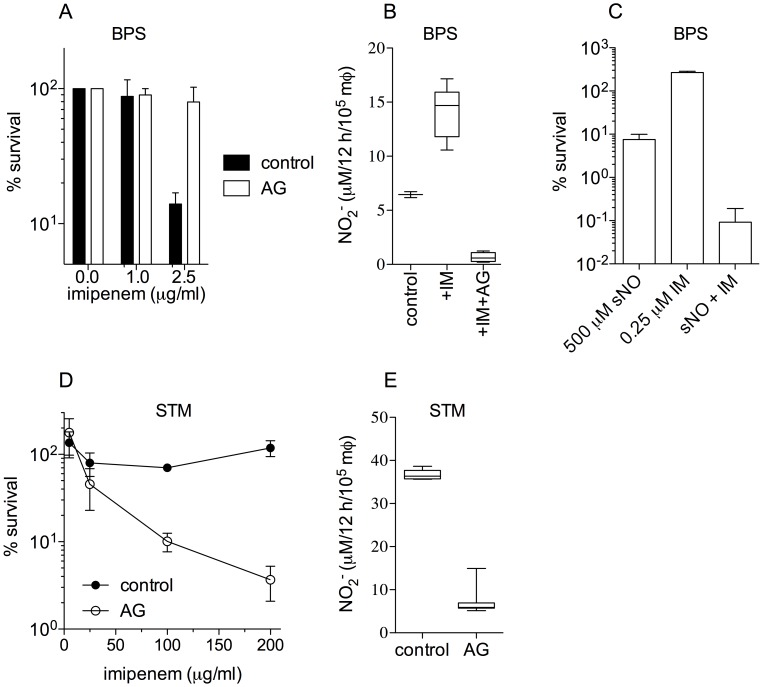
Modulation of antimicrobial activity of imipenem by NO produced by IFNγ-activated J774 cells. Killing of *B. pseudomallei* (BPS) and *Salmonella* (STM) in IFNγ-activated J774 cells treated with increasing concentrations of imipenem (A and D, respectively). Where indicated, 500 µM of the iNOS inhibitor aminoguanidine (AG) was added to the cells. NO_2_^−^ produced by the macrophages was estimated by the Griess reaction (B and E). Panel C shows the killing of log phase *B. pseudomallei* treated for 2.5 h in LBG broth with 500 µM spermine NONOate (sNO) and/or 0.25 µg/ml imipenem (IM).

Our investigations indicate that chemically-generated NO protects *B. pseudomallei*, *S. enterica* and *E. coli* against the antimicrobial activity of β-lactams. Next, we studied whether NO generated through the enzymatic activity of NO synthases modulates the antimicrobial activity of imipenem. J744 macrophage-like cells were stimulated overnight with 200 U/ml recombinant murine IFNγ. The macrophages were infected with *B. pseudomallei* at an MOI of 4. The data shown in figure 6A indicate that NO produced by IFNγ-treated macrophages enhances the antimicrobial activity of 2.5 µg/ml imipenem. Our investigations identify NO as the mechanism by which recombinant IFNγ enhances the antimicrobial activity of imipenem *in vivo* [37]. These findings, however, contrast with the protective effects observed for NO *in vitro*. Because *B. pseudomallei* are hypersusceptible to NO [30], we tested the survival of *B. pseudomallei* after exposure to a bactericidal concentration of NO and a sublethal amount of imipenem. About 90% of *B. pseudomallei* in the cultures were killed after exposure to 500 µM spermine NONOate, whereas controls treated with 0.25 µM imipenem doubled in cell number during the 2.5 h of the experiment (figure 6C). However, the simultaneous addition of 500 µM spermine NONOate and 0.25 µM imipenem killed ∼99.9% of *B. pseudomallei* in the cultures. Together, these investigations suggest that β-lactams can potentiate the antimicrobial activity of host defenses such as NO. We next tested whether NO produced by IFNγ-treated macrophages can modify the killing of intracellular *Salmonella* by imipenem. In contrast to *B. pseudomallei* (figure 6A), NO produced by IFNγ-treated macrophages appears to protect *Salmonella* against this β-lactam (figure 6D and E), since imipenem killed *Salmonella* in a concentration-dependent manner in macrophages treated with the iNOS inhibitor aminoguanidine.

## Discussion

Our investigations indicate that the antimicrobial activity of β-lactams can be modified by NO produced chemically or enzymatically in the inflammatory response of IFNγ-activated macrophages. At sublethal concentrations, NO protects *B. pseudomallei*, nontyphoidal *Salmonella* and *E. coli* from the antimicrobial activity of β-lactams, and NO generated by IFNγ-activated macrophages shields intracellular *Salmonella* from the cytotoxicity of imipenem. At higher concentrations, however, the bactericidal activity of NO itself against *B. pseudomallei* was potentiated by sublethal concentrations of imipenem.

NO has been proposed to protect bacteria against different classes of antibiotics by promoting antioxidant defenses [18]. However, our investigations suggest that the mechanism by which NO induces resistance of *B. pseudomallei* and other Gram-negative bacteria to β-lactam antibiotics is mediated through the collapse of the PMF. The following independent lines of evidence support the proposed model. First, the concentrations of NO that protect *B. pseudomallei* against β-lactams also inhibit respiratory activity. Second, high throughput sequencing of a transposon library revealed that *B. pseudomallei* mutants in cytochrome oxidase are hyperresistant to β-lactam killing. Third, cyanide, a classical inhibitor of cytochromes, inhibits respiration and protects *B. pseudomallei*, *E. coli* and *S. enterica* against β-lactams. Fourth, the degree of the PMF appears to be inversely associated with the extent of β-lactam-mediated killing. Fifth, dissipation of ΔH^+^ and ΔΨ components of the PMF independently protect against β-lactams. And sixth, killing by imipenem is marginal in anaerobic *B. pseudomallei* cultures, unless the electron transport chain is energized with terminal electron acceptors such as NO_3_^−^. Collapse of the PMF can be added to β-lactamases, mutated penicillin-binding proteins and efflux pumps as strategies that protect bacteria against β-lactam antibiotics. The immediate antibiotic tolerance elicited in response to NO may provide a window of time required for the acquisition of mutations that mediate inheritable resistance to antibiotics.

NO, KCN, CCCP and valinomycin blocked the imipenem-mediated killing of *B. pseudomallei*, *E. coli* and *S. enterica*. These findings indicate that the antimicrobial activity of β-lactams requires an energized membrane. Considering the high affinity of NO for terminal cytochromes of the electron transport chain and the advantage afforded by transposons in cytochrome c or cytochrome c oxidase for the survival of *B. pseudomallei* in the presence of imipenem, we propose that terminal cytochromes are the likely molecular switch by which NO induces tolerance to β-lactams. This model is independently supported by the fact that the concentrations of NO that elicited antibiotic tolerance in *B. pseudomallei* also inhibited O_2_ consumption. Repression of respiratory activity has already been shown to mediate NO-induced resistance of *Salmonella* to aminoglycosides [19]. Our investigations with *Salmonella* and *E. coli* indicate that the ability of NO to inhibit respiration is necessary but not sufficient for β-lactam drug tolerance. Ultimately, the ability of NO to induce antibiotic tolerance seems to be associated with the degree of inhibition of the PMF. For example, NO-dependent tolerance to imipenem in *E. coli* and *Salmonella* grown in EG medium or LBG broth is inversely proportional to the PMF of the bacteria. Interestingly, NO prevented most of the antibiotic activity of imipenem against the strict aerobe *B. pseudomallei* under growth conditions that failed to protect *Salmonella* or *E. coli*. This is likely explained by the fact that the PMF in *B. pseudomallei* is preferentially maintained by the enzymatic activity of cytochrome oxidases. The heavy dependence of *B. pseudomallei* on cytochrome oxidases to maintain the PMF may explain why β-lactam antibiotics are most efficient during the acute phase of therapy at a time when, in the absence of abscesses and NO-mediated immunity, cytochrome oxidases are expected to be fully functional.

The NO blockage of energy-dependent drug uptake has been shown to protect *Salmonella* against aminoglycosides [19]. In an analogous fashion, NO could protect bacteria by interfering with the expression or function of the Omp38 porin that is required to transport β-lactams into the periplasmic space [38]. Blockage of drug uptake, however, may not explain why NO protects bacteria against β-lactam antibiotics, because *B. pseudomallei* treated simultaneously with NO and imipenem yielded even smaller colonies than NO-treated controls. Moreover, NO protected *B. pseudomallei* already exposed to imipenem. The NO-induced tolerance to β-lactams could be mediated by the negative impact that the collapse of the PMF has on metabolism and membrane function. At least three mechanisms could explain how the collapse of the PMF by NO lessens the antibiotic activity of β-lactam drugs. First, electrochemical gradients energize the transport of muropeptides across the cytoplasmic membrane [39]; thus, the negative impact of NO on the PMF may inhibit peptidoglycan biosynthesis. Second, rapid β-lactam-induced lysis requires successful assembly of the divisome [40], [41], an event that is initiated by the PMF-dependent localization of FtsA to the FtsZ septal ring [42]. FtsA serves as a scaffold for the assembly of several morphogenetic proteins, including penicillin-binding proteins [43] that are the targets of β-lactam antibiotics. Consequently, the NO-dependent inhibition of the PMF could delocalize morphogenetic proteins from the division septum, thereby contributing to resistance to β-lactams. Disassembly of the division ring could be a critical step by which NO protects rapidly growing bacteria from β-lactams, but may be of lesser importance in non-dividing stationary phase bacteria. And third, NO and the other chemical inhibitors of the electron transport chain could protect bacteria by stalling growth. According to this idea, imipenem exerted negligible antimicrobial activity against stationary phase *Salmonella*. However, inhibition of cell growth might not explain why sublethal concentrations of NO protect stationary phase *B. pseudomallei* against imipenem. We found it remarkable that the 1,000-fold reduction in viability of nongrowing *B. pseudomallei* was abrogated in the presence of sublethal concentrations of NO.

NO did not select for intrinsically resistant populations, because the surviving bacteria remained susceptible to imipenem. Constant NO fluxes were required for the elicitation of tolerance to imipenem, and the effects were short-lived. The inhibition of metal centers in terminal oxidases of the electron transport chain, with the consequent reduction in PMF, provides a reasonable model for the fast and transient adaptation of *B. pseudomallei, E. coli* and *Salmonella* to β-lactam antibiotics. The transient protection afforded by NO can be better understood if we consider that the *k*_off_ value for the dissociation of NO from cytochrome *aa*_3_ is 0.01 sec^−1^ [44]. Thus, the association of cytochrome *c* oxidase to NO would last about 1 min (i.e., *t*_1/2_ of 69 sec). In other words, the fast denitrosylation of metal prosthetic groups in the terminal cytochromes of the electron transport chain could explain the transient protection noted after NO is removed from the bacterial cultures.

Oxidative stress has been proposed as a common killing mechanism of bactericidal antibiotics [21], and the antioxidant defenses elicited by NO are thought to mediate resistance of *B. subtilis* to several classes of antibacterials [18]. Our investigations indicate that NO prevents antibiotic killing despite increasing the rate of H_2_O_2_ synthesis. The stasis of electrons that follows the nitrosylation of terminal cytochrome oxidases of the electron transport chain can facilitate the adventitious reduction of O_2_ by flavin cofactors or Q sites of NADH dehydrogenases [45], [46]. The O_2_^−^ generated in this process spontaneously or enzymatically dismutates to H_2_O_2_. This model may explain why imipenem, similar to other β-lactam antibiotics that increase bacterial respiratory rates [22], diminishes H_2_O_2_ synthesis. The increased H_2_O_2_ synthesis noted in NO-treated bacteria challenges the notion that NO enhances antibiotic resistance by eliciting antioxidant defenses [18]. Our investigations are consistent with recent work that has reported that bactericidal antibiotics can kill microorganisms in the absence of oxidative stress [22], [23]. This idea is further substantiated by the fact that imipenem can kill *B. pseudomallei* in anaerobic cultures given that the bacteria are actively respiring the terminal electron acceptor NO_3_^−^.

Although sublethal concentrations of NO reversed β-lactam-mediated killing of *B. pseudomallei*, NO produced by IFNγ-primed macrophages synergized with imipenem in killing intracellular *B. pseudomallei*. Our investigations identify NO as the mechanism by which recombinant IFNγ enhances the antimicrobial activity of imipenem *in vivo* [47]. We find it remarkable that *inos*, which is just one of over 150 loci regulated by IFNγ [48], made such a difference in the outcome of imipenem treatment. Cumulatively, these investigations support the widely accepted concept that immunocompetent hosts respond better to antibiotic therapy than immunodeficient controls. A closer look at our investigations indicate, however, that imipenem potentiates the killing activity of NO *in vivo* and not *vice versa*. In fact, sublethal concentrations of imipenem augmented the bactericidal activity of NO in an exponential fashion.

Weakening of the cell wall by β-lactams increases NO-mediated killing. This observation could be explained by a model in which the outstanding killing that NO exerts against *B. pseudomallei* is a direct consequence of membrane dysfunction. Because *B. pseudomallei* draw most energy from oxidative phosphorylation, nitrosylation of terminal cytochromes of the electron transport chain could have greater deleterious actions on the energetics and membrane function of the aerobe *B. pseudomallei* as compared to more metabolically pliable organisms such as *Salmonella* that can draw significant energy from fermentation. Degree or duration of the inhibition of the respiratory chain by NO could then rationalize why sublethal concentrations of NO protect against β-lactams, whereas higher NO fluxes become more lethal in the presence of imipenem. The clinical relevance of these findings can already be inferred from the observation that mice treated with recombinant IFNγ and ceftazidime clear acute *B. pseudomallei* infections [37]. It remains puzzling that during the natural course of meliodosis ceftazidime is largely ineffective during the eradication phase of treatment. Reduced respiratory activity imposed by growing abscesses or the sublethal amounts of NO could contribute to the more limited use of β-lactams in the eradication phase of therapy.

## Supporting Information

Figure S1 Functional categorization of *B. pseudomallei* essential genes.
(TIF)

Figure S2 Effect of valinomycin on the PMF as estimated fluorometrically by measuring the accumulation of DiSC_3_(5).
(TIFF)

Table S1 List of *B. pseudomallei* essential genes.
(PDF)
