# Supplementary figures and images for: Nitric Oxide from IFNγ-Primed Macrophages Modulates the Antimicrobial Activity of β-Lactams against the Intracellular Pathogens Burkholderia pseudomallei and Nontyphoidal Salmonella
Source: PLoS Negl Trop Dis. 2014 Aug 14;8(8):e3079. doi: 10.1371/journal.pntd.0003079 (PMC4133387; doi:10.1371/journal.pntd.0003079)

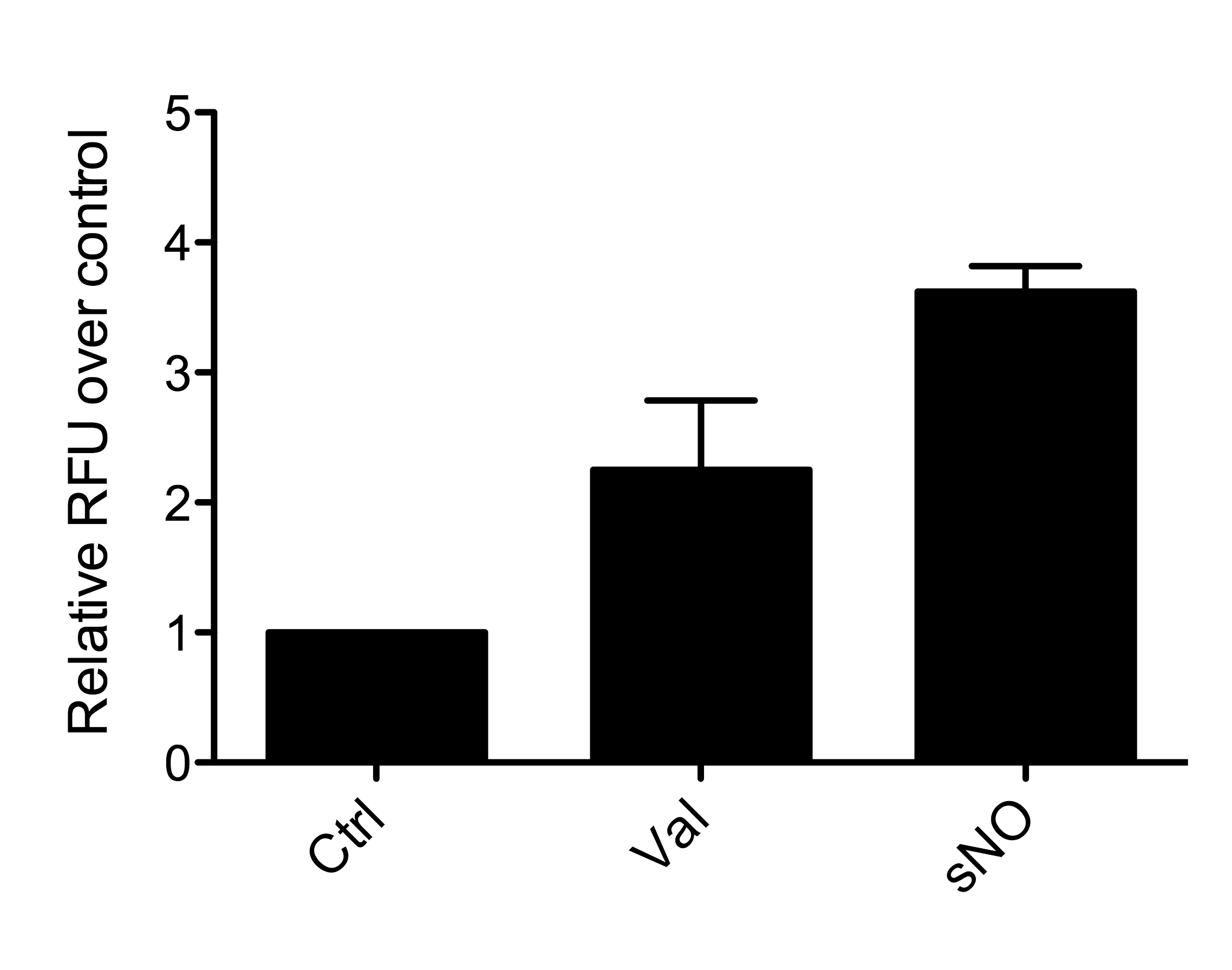

Supplement: Figure S2 — Effect of valinomycin on the PMF as estimated fluorometrically by measuring the accumulation of DiSC3(5). (TIFF) [file pntd.0003079.s002.tiff]
