# Supplementary material for: Nitric Oxide from IFNγ-Primed Macrophages Modulates the Antimicrobial Activity of β-Lactams against the Intracellular Pathogens Burkholderia pseudomallei and Nontyphoidal Salmonella
Source: PLoS Negl Trop Dis. 2014 Aug 14;8(8):e3079. doi: 10.1371/journal.pntd.0003079 (PMC4133387; doi:10.1371/journal.pntd.0003079)
Supplement: Table S1 — List of B. pseudomallei essential genes. (PDF) [file pntd.0003079.s003.pdf]

List of B. pseudomallei essential genes

atpB
atpC
atpH
BPSL0458
benD
BPSS0046
BPSS1886
BPSS2159
BPSL1215
BPSL1217
BPSS1947
nuoL
nuoN
BPSL0395
BPSL0666
eno
gltA
gmhD
pgm
waaE
BPSL0697
BPSL1203
BPSL1219
BPSL1454
BPSL2463
BPSL2476
BPSS0553
eda
etfA
etfB
fdsD
fdxA
metK
nuoA
ppa
tauD
udg
nuoB
nuoD
nuoF
nuoH
nuoJ
nuoM
sdhA
ureB
ipk
mgsA
pchA
BPSS1719
BPSS1720
icd
mdh
sucA

sucB
asd
astB
BPSL2847
BPSL2925
catB
cysM
dapA
dapB
dapD
dapE
folD
glyA
iscS
pyrH
soxD
BPSL0184
BPSL0185
BPSL0514
BPSL1472
BPSS1219
ddl
engB
ftsA
ftsL
ftsQ
ftsW
ftsZ
minE
mraY
murB
murC
murD
murE
murF
murG
oxa
BPSL0225
BPSL1899
BPSS2185
BPSS2186
flgI
fliR
BPSS0814
BPSS1872
BPSS1874
BPSL3120
BPSS2349
gyrB
BPSL3405
BPSS2350
BPSL0397
BPSL0461
BPSL0462
BPSL2893

BPSL3035
coaD
dfp
folC
hemC
hemE
hemF
hemH
hemL
ubiB
BPSL2960
cobT
dxs
pdxA
ribD
ribE
thiE
thiG
thiS
BPSL0832
BPSL0966
BPSL1437
BPSL1459
BPSL1979
BPSL2992
BPSS2351
dnaA
dnaB
dnaE
dnaG
dnaJ
dnaN
dnaQ
dnaX
holA
ligA
nrdB
rnc
rnpA
ruvA
ruvB
ssb
xseB
accA
accB
accD
acpP
BPSL0211
BPSL0615
BPSL0878
BPSL1199
BPSL2154
BPSL2247
BPSL2443
BPSL2961

BPSS0034
BPSS0232
BPSS0621
BPSS1277a
BPSS1445
BPSS1446
BPSS1447
BPSS2033
BPSS2257
BPSS2340
dxr
fabD
fabF
fabI
fabZ
ispB
ispD
ispF
ispG
kdsB
lipA
lipB
lpxA
lpxB
lpxC
rmlC
uppS
waaA
bsaR
bsaT
bsaV
BPSL2123
adk
apaH
BPSL1162
cmk
dcd
guaB
prs
purA
purB
purE
purK
purL
pyrD
pyrG
thyA
tmk
alaS
BPSL2240
BPSL2260
cca
fmt
glnS
glyQ

glyS
hemA
leuS
lysS
metG
pheT
proS
serS
thrS
tyrZ
valS
BPSL0158
BPSL0159
BPSL0162
BPSL0166
BPSL0168
BPSL0574A
BPSL1251
BPSL3351
BPSS1047
BPSS1067
BPSS1068
BPSS1069
BPSS1070
BPSS1073
BPSS1079
BPSS1086
BPSS1086a
ihfA
bicP
BPSL0119
BPSL0918
BPSL1516
BPSL2829
BPSL2909
BPSS1432
hscA
hscB
trxA
trxB
BPSL2185
BPSL2199
lepB
lspA
BPSS0505
nusA
nusB
nusG
rho
rpoA
rpoB
rpoC
BPSL0128
BPSL1658a
BPSL2303

BPSL3115
BPSL3281
BPSS0187
BPSS0565
BPSS0796A
BPSS1391
BPSS1824
BPSS1890
BPSS1908
BPSS2204
BPSS2231
hfq
lexA
argS
aspS
BPSL1355
BPSS1195
BPSS1812
BPSS1813
cysS
ffh
frr
gatA
gatB
gatC
gcp
infB
infC
prfA
prfB
rbfA
rplA
rplB
rplC
rplD
rplE
rplF
rplJ
rplK
rplM
rplN
rplO
rplP
rplQ
rplR
rplS
rplT
rplU
rplV
rplW
rplX
rpmB
rpmD
rpmE2
rpmF

rpmH
rpmI
rpmJ
rpsB
rpsC
rpsD
rpsE
rpsF
rpsG
rpsH
rpsI
rpsJ
rpsK
rpsL
rpsM
rpsN
rpsO
rpsP
rpsQ
rpsS
tsf
BPSL0672
era
rimM
amrA
amrB
BPSL0438
BPSL0534
BPSL0535
BPSL0658
BPSL0963
BPSL0964
BPSL0978
BPSL1118
BPSL1779
BPSL1783
BPSL1784
BPSL1802
BPSS0243
BPSS0366
BPSS0879
BPSS1613
BPSS1618
BPSS1622
BPSS1623
BPSS1625
BPSS1626
BPSS1628
BPSS1629
BPSS1939
BPSS2325
bsaP
fur
hmuV
lolB

lolD
mscL
nuoC
nuoK
phnL
sctQ
sctS
secA
secD
secE
secF
secG
tatB
yajC
BPSL0335
BPSL0398
BPSL0677
BPSL0860
BPSL0872
BPSL0874
BPSL0877
BPSL1200
BPSL1471
BPSL2287
BPSL2965
BPSL3059
BPSL3069
BPSL3274
BPSS0156
BPSS0329
BPSS0383
BPSS0738
BPSS0760
BPSS0792
BPSS1343
BPSS1811
BPSS2068
BPSS2264
BPSS2310
BPSL0310
BPSL0536
BPSL1193
BPSL1298
BPSL2466
BPSL2508
BPSL2703
BPSL3146
BPSS0289
BPSS0453
BPSS0454
BPSS0629
BPSS0658
BPSS0756
BPSS1024
BPSS1038

BPSS1506
BPSS1578
BPSS1647
BPSS1743
BPSS1772
BPSS1876A
BPSS2107
BPSS2232
BPSS2237
BPSL0339
BPSL2277
BPSL2937
BPSS0170
BPSS2224
BPSL0721
BPSL1194
BPSL1320
BPSL1473
BPSL1474
BPSL1859
BPSL1901
BPSL2150
BPSL2180
BPSL2934
BPSL3091
BPSL3149
BPSL3240
BPSL3254A
BPSS0020
BPSS0152
BPSS0592
BPSS0597
BPSS1338
BPSS1378
BPSS1661
BPSS1703
BPSS1907
BPSS2194
BPSS2241
omlA
BPSL0089
BPSL0317
BPSL0332
BPSL0537
BPSL0588
BPSL0635
BPSL0639
BPSL0802
BPSL0870
BPSL0975
BPSL1133
BPSL1147
BPSL1255
BPSL1434
BPSL1468

BPSL1601
BPSL1766
BPSL2048
BPSL2064
BPSL2088
BPSL2170
BPSL2283
BPSL2288
BPSL2334
BPSL2345
BPSL2507A
BPSL2576
BPSL2645
BPSL2664
BPSL2779
BPSL2819
BPSL2864
BPSL3145
BPSS0007
BPSS0080a
BPSS0177
BPSS0184
BPSS0211
BPSS0390
BPSS0416A
BPSS0480
BPSS0518
BPSS0527
BPSS0528
BPSS0573
BPSS0599
BPSS0636
BPSS0649
BPSS0676
BPSS0682
BPSS0753
BPSS0769A
BPSS0795
BPSS0895
BPSS0961
BPSS1003
BPSS1094
BPSS1138
BPSS1185a
BPSS1330
BPSS1345
BPSS1383
BPSS1398
BPSS1399
BPSS1406
BPSS1480
BPSS1505
BPSS1507
BPSS1517
BPSS1612

BPSS1645
BPSS1655
BPSS1658
BPSS1716
BPSS1814
BPSS1817
BPSS1877
BPSS1972
BPSS1981
BPSS2001
BPSS2006
BPSS2019
BPSS2020
BPSS2023
BPSS2101
BPSS2131a
BPSS2144
BPSS2179
BPSS2181
BPSS2215
BPSS2225
BPSS2227
BPSS2292
BPSS2297
BPSS2298
BPSS2338
BPSL0240
BPSL1469
BPSL1584
BPSL1673
BPSL2767
BPSS0084
BPSS0189
BPSS0402B
BPSS0653
BPSS0728
BPSS1207
BPSS1385A
BPSS1449a
BPSS1728A
BPSS1774A
BPSS2313
